# Concordance of SVR12, SVR24 and SVR durability in Taiwanese chronic hepatitis C patients with direct-acting antivirals

**DOI:** 10.1371/journal.pone.0245479

**Published:** 2021-02-04

**Authors:** Chuan-Pin Lin, Po-Cheng Liang, Ching-I Huang, Ming-Lun Yeh, Po-Yao Hsu, Cheng-Ting Hsu, Yu-Ju Wei, Ta-Wei Liu, Ming-Yen Hsieh, Nai-Jen Hou, Tyng-Yuang Jang, Yi-Hung Lin, Chih-Wen Wang, Zu-Yau Lin, Shinn-Cherng Chen, Chung-Feng Huang, Jee-Fu Huang, Chia-Yen Dai, Wan-Long Chuang, Ming-Lung Yu

**Affiliations:** 1 Hepatobiliary Division, Department of Internal Medicine, Kaohsiung Medical University Hospital, Kaohsiung Medical University, Kaohsiung, Taiwan; 2 Faculty of Internal Medicine and Hepatitis Research Center, School of Medicine, College of Medicine, and Center for Cancer Research and Liquid Biopsy, Kaohsiung Medical University, Kaohsiung, Taiwan; 3 Department of Internal Medicine, Kaohsiung Municipal Ta-Tung Hospital, Kaohsiung, Taiwan; 4 Department of Internal Medicine, Pingtung Hospital, Ministry of Health and Welfare, Ping-Tung, Taiwan; 5 Department of Internal Medicine, Kaohsiung Municipal Siaogang Hospital, Kaohsiung, Taiwan; 6 Department of Occupational Medicine, Kaohsiung Medical University Hospital, Kaohsiung Medical University, Kaohsiung, Taiwan; 7 Insitute of Biomedical Sciences, National Sun Yat-Sen University, Kaohsiung, Taiwan; 8 Center For Intelligent Drug Systems and Smart Bio-devices (IDS^2^B) and Department of Biological Science and Technology, College of Biological Science and Technology, National Chiao Tung University, Hsin-Chu, Taiwan; 9 Center for Lipid Science and Aging Research Center, Kaohsiung Medical University, Kaohsiung, Taiwan; Cornell University, UNITED STATES

## Abstract

**Background/Aims:**

Undetectable HCV RNA 12 weeks after the end of treatment (SVR12) has been the valid efficacy endpoint in the era of direct-acting antivirals (DAAs). Its concordance with SVR4 and SVR24 and long-term durability is unknown in Taiwanese chronic hepatitis C (CHC) patients.

**Methods:**

A total of 1080 CHC patients who received all-oral DAAs and an achieved end-of-treatment virological response (EOTVR), defined as undetectable HCV RNA at the end of therapy, were consecutively enrolled. HCV RNA was monitored 4, 12, and 24 weeks after EOT. Patients who achieved SVR24, defined as undetectable HCV RNA 24 weeks after EOT, were followed annually for assessing SVR durability.

**Results:**

Eleven (1.02%) patients experienced HCV RNA reappearance after EOT. The most frequent timing of RNA reappearance was observed at SVR4 (n = 7), followed by SVR12 (n = 3) and SVR 24 (n = 1). The positive predictive value (PPV) and negative predictive value (NPV) of SVR4 in predicting SVR12 were 99.7% and 100%, respectively, whereas the PPV and NPV of SVR12 in predicting SVR24 were 99.9% and 100%, respectively. Pyrosequencing confirmed delayed relapse rather than reinfection for the patient who had detectable HCV RNA at SVR24. Among 978 patients who achieved SVR24, after a median follow-up period of 17.3±8.2 months, the SVR durability is 100% up to a 4-year follow-up.

**Conclusion:**

Achievement of SVR12 provides excellent durability of HCV seroclearance after DAA therapy. On-demand HCV RNA beyond SVR12 should be recommended for patients with unexplainable abnormal liver function or high-risk behaviors.

## Introduction

Hepatitis C virus (HCV) infection is one of the leading etiologies of liver-related morbidities and mortalities worldwide [1]. Approximately 71 million individuals are chronically infected with HCV worldwide [2], accounting for one-third of the patient population with hepatocellular carcinoma (HCC). Fortunately, HCV eradication by antiviral therapy greatly reduces the risk for HCC, liver decompensation and liver-related mortality [3–6].

The definition of successful HCV eradication is a sustained virological response (SVR), which is defined as undetectable HCV RNA throughout 24 weeks of the posttreatment follow-up period (SVR24) in the interferon era [7–9]. Thanks to their excellent efficacy and tolerability [1, 10], all-oral direct-acting antiviral agents (DAAs) have been the standard of care for chronic hepatitis C (CHC) since 2014. Due to the high concordance of SVR24 and SVR12, defined as undetectable HCV RNA throughout the 12-week posttreatment follow-up period, SVR12 has been widely accepted as a valid efficacy endpoint in the DAA era [11]. However, the concordance between SVR12 and SVR24 has rarely been systematically assessed in Taiwanese CHC patients. Notably, there were few cases of late recurrent viremia, defined as patients who achieved SVR12 but experienced HCV RNA recurrence at or after follow-up week 24 [12]. It is important to distinguish late virologic relapse from reinfection in view of the post-DAA follow-up strategy. On the other hand, a high SVR durability has been reported for patients who have successfully eradicated HCV by daclatasvir-based regimens [13]. While the result is consistent with the use of recently approved and more potent DAAs, it remains elusive in Taiwanese patients. Overall, the current study aimed to identify the timing of virological failure among CHC patients who achieved end-of-treatment virological response (EOTVR) by DAAs. Accordingly, we also sought to address the concordance among SVR4, SVR12, and SVR24, as well as long-term SVR durability, in a well-characterized CHC cohort.

## Methods

CHC patients who received all-oral DAA regimens in the outpatient departments of Kaohsiung Medical University Hospital and two regional hospitals, Kaohsiung Municipal Ta-Tung Hospital and Kaohsiung Municipal Siaogang Hospital, were consecutively enrolled during the daily practice from February 2015 to October 2018. The treatment regimens and strategies conformed to the regional guidelines and the regulation of the Health and Welfare Department of Taiwan [1] and regional guidelines [14, 15]. The inclusion criteria were as follows: (1) adults aged 20 years or older; (2) those who were interferon treatment-naïve or treatment experienced; and (3) those who completed the full treatment course and achieved the end-of-treatment virological response (EOTVR, defined as HCV RNA seronegativity at the end of treatment). Patients were excluded if they had the following: (1) HCV RNA seropositivity at EOT, either due to treatment failure or virological breakthrough, or (2) lost to follow-up during the treatment or follow-up period.

Patients who had virological data at posttreatment week 4 (SVR 4), posttreatment week 12 (SVR 12), and posttreatment week 24 (SVR 24) were evaluated for the concordance of SVR4 with SVR 12 and SVR12 with SVR 24. Among the patients who achieved SVR24, HCV RNA was followed annually to assess SVR durability. The study was approved by the institutional review board of Kaohsiung Medical University Hospital (IRB number: KMUHIRB-F(I)-20170053), which conformed to the guidelines of the International Conference on Harmonization for Good Clinical Practice. All patients provided written informed consent.

The primary objective was to address RNA reappearance after EOT. The HCV genotype was assessed before treatment and at the time of RNA reappearance. HCV RNA and genotypes were determined using a real-time PCR assay (RealTime HCV; Abbott Molecular, Des Plaines IL, USA; detection limit: 12 IU/ml) [16]. For patients who had RNA reappearance after SVR12, viral sequencing on nonstructural protein 3 (NS3) and NS5B was performed to compare the viral genetic similarity (AnGene Biotechnology Co., Ltd.; targeting the core protein). Liver cirrhosis was defined by liver histology, transient elastography (FibroScan®; Echosens, Paris, France; > 12 kPa) [17], acoustic radiation force impulse (> 1.98 m/s) [18], or fibrosis-4 index (>6.5); the presence of clinical, radiologic, endoscopic, or laboratory evidence of cirrhosis and/or portal hypertension; or symptoms of clinical hepatic decompensation (ascites, hepatic encephalopathy, jaundice, or variceal hemorrhage).

### Statistical analyses

Group means are presented as the means ± standard deviations. The fibrosis index 4 (FIB-4) was calculated by the following formula: age (years) × aspartate aminotransferase (AST) [U/L]/(platelets [10^9^/L] [8] × (alanine transaminase (ALT) [U/L]))^1/2^. The concordance of the virological responses between different time points (SVR4, SVR12 and SVR24) was compared and expressed as positive predictive value (PPV) and negative predictive value (NPV). The procedures were performed using the SPSS 22.0 statistical package (SPSS Inc., Chicago, IL).

## Results

**Fig 1:**
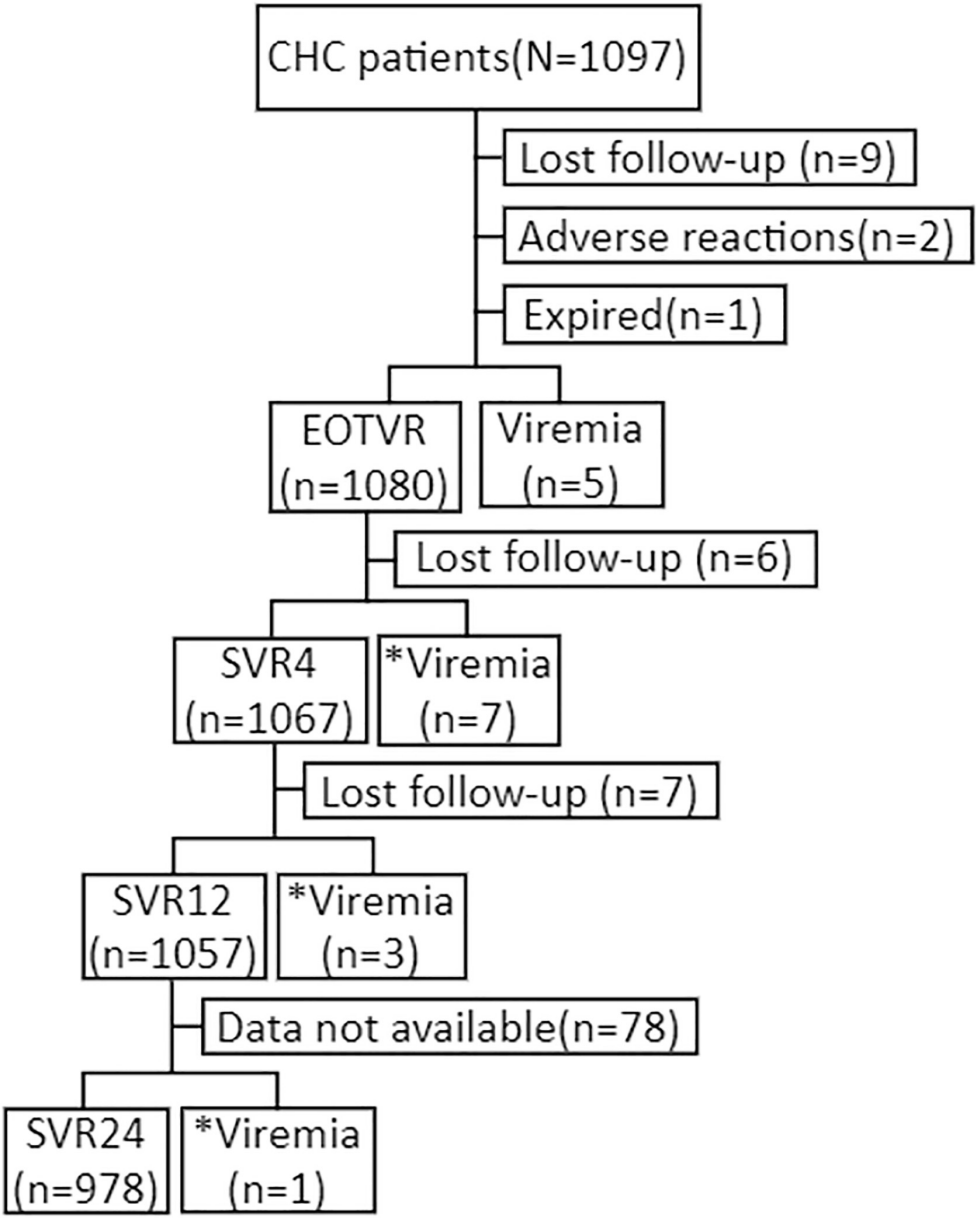
Study flowchart. Abbreviations: CHC: chronic hepatitis C. EOTVR: end-of-treatment virological response. * Viremia represents patients who experienced virological reappearance after EOT.

A total of 1097 patients were initially enrolled. After excluding patients who were lost to follow-up (n = 9), early treatment termination (n = 2), mortality during treatment (n = 1), and remained viremic at EOT (n = 5), 1080 patients who achieved EOTVR were enrolled for final analysis in the current study (Fig 1). The basic demographic, virological, and clinical features and DAA regimens of the patients are shown in Table 1. The mean age was 62.9 years. Males accounted for 43.0% of the population. The majority of patients had HCV genotype 1 infection (HCV-1, 69.7%). Five hundred sixty-five patients (52.3%) had liver cirrhosis. The mean FIB-4 was 4.3. The most commonly used DAA was paritaprevir/ritonavir/ombitasvir/dasabuvir (PrOD, 37.3%), followed by sofosbuvir/ledipasvir (17.3%).

**Table 1:** Basic characteristics and treatment regimens of the patients.

	N = 1080
Gender, male/female, n (%)	464/616 (43.0/57.0)
Age, years (mean±SD)	62.9±11.2
Platelet count, x1000 /mm3 (mean±SD)	161±71
AST, IU/L (mean±SD)	73.7±51.1
ALT, IU/L (mean±SD)	80.9±64.8
Serum albumin, g/dL(mean±SD)	4.2±0.4
Serum bilirubin, mg/dL (mean±SD)	1.02±0.54
Creatinine, mg/dL(mean±SD)	1.08±1.28
HCV viral load, 1og IU/ml (mean±SD)	6.40±7.18
High HCV viral loads, n (%)	510 (47.2)
HCV genotype, n (%)	
1	753 (69.7)
Non-1	327 (30.3)
Liver cirrhosis, n (%)	565 (52.4)
HIV co-infection, n (%)	34(3.1)
Persons who inject drugs, n (%)	58(5.4)
DAA regimen, n (%)	
PrOD±RBV	403 (37.3)
DCV/ASV±RBV	59 (5.5)
SOF/RBV	169 (15,6)
SOF/LDV±RBV	187 (17.3)
SOF/DCV±RBV	110 (10.2)
ELB/GRZ	115 (10.6)
GLE/PIB	32(3.0)
SOF/VEL	5 (0.5)

Note: AST, aspartate aminotransferase. ALT, alanine aminotransferase. PrOD, Paritaprevir/ritonavir/Ombitasvir/Dasabuvir. DCV, Daclatasvir. ASV, Asunaprevir. SOF, Sofosbuvir. LDV, Ledipasvir. ELB, Elbasvir. GRZ, Grazoprevir. GLE, Glecaprevir. PIB, Pibrentasvir. VEL, Velpatasvir. RBV, Ribavirin. HIV, human immunodeficiency virus.

Among the subjects, 11 (1.02%) patients experienced virological reappearance, and the most frequent timing of viral reappearance was week 4 (n = 7), followed by week 12 (n = 3) and week 24 (n = 1) after EOT. Of the 1060 patients with SVR12 data available, 1057 (99.7%) achieved an SVR12, leading to a 99.7% PPV and a 100% NPV of SVR4 for SVR12. Of the 979 patients with SVR24 data available, 978 (99.9%) achieved an SVR24, leading to a 99.9% PPV and a 100% NPV of SVR12 for SVR24.

The characteristics and serial viral kinetics of the eleven patients who encountered HCV RNA reappearance after EOT are listed in Table 2. Among them, six (54.5%) were infected with HCV genotype 2 and received a sofosbuvir-plus-ribavirin regimen. Nine patients (81.8%) had liver cirrhosis. All HCV genotypes at the time of RNA reappearance were the same as the pretreatment genotypes. A 72-year-old cirrhotic female patient (Case 11) who had RNA reappearance at SVR24 also had the same HCV genotype, HCV-1b, before and after DAA treatment. Pyrosequencing revealed 99.7% similarity before treatment and at the time of RNA reappearance, indicating virological relapse rather than reinfection of the patient.

**Table 2:** Characteristics and serial viral kinetics of the patients with virological failure.

Case Number	Age/gender	BL genotype	BL HCV viral load (*10^3^IU/ml)	Regimen/Weeks of Tx	LC	W2	W4	W8	EOT	M1	M3	M6
1	61/M	2	48.2	SOF+RBV/12	+	0.06	<0.03	<0.03	<0.03	<0.03	110.7	NA
2	61/F	1b	1787.3	DCV+ASV/24	-	0.06	<0.03	LLOD	LLOD	LLOD	1500.4	929.9
3	65/F	2	521.4	SOF+RBV/12	+	0.16	0.06	<0.03	LLOD	1692.3	457.7	67.2
4	66/M	2	3918.8	SOF+RBV/12	+	<0.03	LLOD	LLOD	LLOD	340.5	352.4	217.5
5	46/M	2	1677.5	SOF+RBV/12	-	0.10	<0.03	LLOD	LLOD	0.1	3305.2	4340.0
6	49/F	2	1342	SOF+RBV/12	+	0.08	LLOD	LLOD	LLOD	4166.3	2288.2	NA
7	58/M	1a	113.8	SOF+LDV/12	+	0.03	<0.03	<0.03	<0.03	122.4	100.5	NA
8	62/M	2	793.6	SOF+RBV/12	+	LLOD	LLOD	LLOD	LLOD	20.0	19.7	NA
9	61/M	1b+2b	507.5	ELB+GRZ/12	+	0.07	<0.03	LLOD	LLOD	LLOD	759.3	586.2
10	42/M	6	9482	SOF+LDV/12	+	<0.03	LLOD	LLOD	LLOD	4709.6	12192.5	16462.7
11	72/F	1b	1219	ELB+GRZ/12	+	0.10	0.05	LLOD	LLOD	LLOD	LLOD	425.2

Note: Abbreviations: M, male. F, female. BL, baseline. Tx, treatment. SOF, Sofosbuvir. RBV, Ribavirin. DCV, Daclatasvir. ASV, Asunaprevir. LDV, Ledipasvir. ELB, Elbasvir. GRZ, Grazoprevir. LLOD: Lower Limit of Detection, NA: Not available.

**Fig 2:**
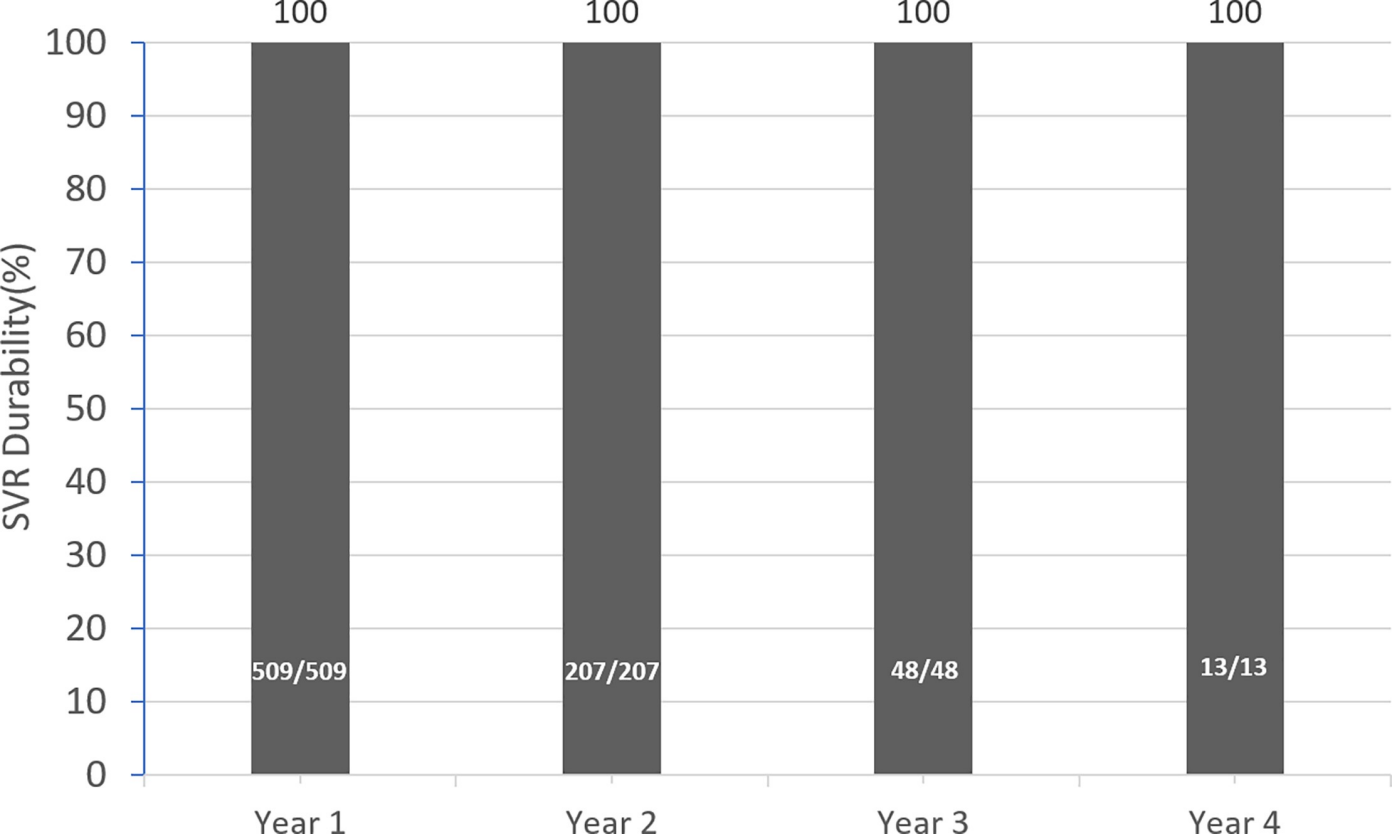
SVR durability beyond SVR24. SVR: sustained virological response.

The mean follow-up period in the post-SVR era was 17.2±7.6 months. The SVR durability among the patients who achieved SVR24 was 100% (509/509), 100% (207/207), 100% (48/48) and 100% (13/13) at year 1, year 2, year 3 and year 4, respectively (Fig 2).

## Discussion

The current study constantly supported the application of SVR12 as the primary efficacy endpoint in the era of DAAs for Taiwanese patients. With the broad use of more potent DAAs, including the pangenotypic glecaprevir/pibrentasvir and sofosbuvir/velpatasvir, the concordance of SVR 4 with SVR 12 was 99.7%, and the concordance of SVR 12 with SVR24 was 99.9%. Only one case experienced late virological relapse at SVR24. In addition, the durability beyond SVR24 was 100% in the current cohort.

Of the patients who relapsed after the end of therapy, two-thirds relapsed within the first 4 weeks, whereas one-third relapsed between posttreatment weeks 4 and 12, indicating that SVR4 may not be a suitable endpoint for primary efficacy analyses. On the other hand, SVR12 and SVR24 were highly concordant, which was in line with previous studies [11, 19]. The current study reinforced the recommendation of regional guidelines [20], which stated that both SVR12 and SVR24 could serve as the endpoints of therapy given > 99% concordance. Reddy et al. reported that twelve out of 1329 patients (0.9%) experienced virological relapse after SVR12 after using daclatasvir-based regimens. Among them, nine relapsed between SVR12 and SVR24, whereas three relapsed after SVR24. Notably, the majority of patients who relapsed after SVR12 had used less potent DAAs (daclatasivir/asunaprevir or interferon-containing regimens) [13]. In the current study, there was only one patient who relapsed at SVR24. With the current use of more potent DAAs, clinicians may have more confidence using SVR12 as the primary efficacy endpoint in the clinical setting.

The SVR durability in patients who achieved SVR24 was 100% beyond 4 years of follow-up in the current study. The result was also similar to that in a long-term follow-up study [13]. The timing and frequency of rechecking HCV RNA in patients who were documented to have an SVR leaves room for discussion [21]. Annual HCV RNA follow-up for reinfection in certain high-risk populations, such as PWID and men who have sex with men, was recommended by regional consensus [15, 20]. Patients who have unexplained abnormal liver function during the follow-up period may also be indicated regardless of the risk of reinfection. Boschi et al. [22] reported a 53-year-old HIV-negative cirrhotic man who was chronically infected with HCV genotype 4r. He was treated with sofosbuvir plus simeprevir for 12 weeks and achieved SVR24. The patient had no injectable drug use or any other potential risk behavior for HCV acquisition. However, unexpectedly detectable HCV RNA was noted 78 weeks after EOT. Phylogenetically clustered protease sequences confirmed the diagnosis of late relapse. The current cohort comprised a subset of patients with HIV coinfection and PWID, and there were no patients who experienced reinfection as of the time of manuscript writing. A longer observational period is warranted to clarify the issue of reinfection or delay relapse in the post-SVR era.

There were some limitations in the current study. Gene sequencing was not performed in patients who had HCV RNA reappearance before SVR12. We could not completely exclude the possibility of reinfection rather than relapse in these patients. However, the HCV genotypes were the same before and after treatment, and there were no known risk factors for reinfection (e.g., PWID, HIV coinfection, and MSM), which favored relapse of these patients. Although the SVR durability was 100% at present, a longer follow-up duration is needed for the potential chance of extremely late relapse or reinfection in the cohort [13]. In conclusion, SVR12 remains the standard endpoint following DAA therapy. The current National Health Insurance Administration (NHIA) of Taiwan supports HCV RNA testing until SVR12. Although the incidence of late recurrence after SVR12 is rare, regular or on-demand follow-up of HCV RNA after SVR 12 may be warranted, particularly for patients with abnormal liver function and high-risk behaviors.

## Supporting information

S1 Dataset (XLSX)

S2 Dataset (XLSX)
